# Effects of Post-Grazing Sward Height and Early or Late Turnout Date to Pasture on the Performance of Dairy Cross-Bred Steers

**DOI:** 10.3390/ani16121790

**Published:** 2026-06-09

**Authors:** Andrew Mc Namee, Denis Mc Crudden, Edward G. O’Riordan

**Affiliations:** 1 Department of Life & Physical Science, Atlantic Technological University, Letterkenny, F92 FC93 Co. Donegal, Ireland; 2 Teagasc, Animal & Grassland Research and Innovation Centre, Grange, Dunsany, C15 PW93 Co. Meath, Ireland

**Keywords:** average daily gain, carcass traits, dairy beef systems, forage intake, grazing strategies, pasture-based systems, sward height, turnout date

## Abstract

Efficient grazing management is critical for optimising animal growth and carcass quality in dairy beef systems; however, the combined effects of turnout date to spring pasture and post-grazing sward height have not been well quantified. This study evaluated the effects of spring turnout date (early vs. late) and post-grazing sward height (3.5 vs. 5.5 cm) on steer performance, intake, and carcass attributes, over three production cycles in a dairy calf-to-beef system. A total of 188 dairy and dairy × beef steers (initial body weight approximately 250–285 kg) were used across three experiments, conducted in a pasture-based calf-to-beef system. Animals grazed for ~200 days followed by ~100 days of indoor finishing. Early turnout improved average daily gain during early- and mid-season (*p* < 0.05 to *p* < 0.001), but differences had disappeared by housing, with no effect on carcass traits (*p* > 0.05). Grazing swards to 5.5 cm increased average daily gain at pasture (*p* < 0.01) and housing weight in the three experiments *(p* < 0.01). Animals grazed to 3.5 cm at pasture subsequently had a higher indoor finishing average daily gain (*p* < 0.05). Grazing to a stubble height of 5.5 cm improved (*p* < 0.001) daily gain at pasture but reduced finishing daily gain (*p* < 0.001). Carcass weight per day of age and were unaffected by either factor *(p* > 0.05), and no significant turnout × post-grazing sward height interactions were detected for any measured variables (*p* > 0.05). These findings indicate that maintaining a higher post-grazing sward height provides improved animal performance, while early turnout offers short-term gains, supporting grazing strategies that prioritise sward height management for sustained performance.

## Simple Summary

This study evaluated how spring turnout date and post-grazing sward height influence steer growth, pasture intake, carcass traits, and meat quality in grass-based dairy–beef systems. Across three experiments, early turnout improved short-term average daily gain during the grazing season but had no lasting effect on carcass traits or meat quality. Grazing to a higher post-grazing sward height of 5.5 cm increased animal liveweight gains at pasture and supported higher pasture intake, while tighter grazing to 3.5 cm was associated with lower gains at pasture but with higher gains while finishing indoors. Carcass and meat quality traits remained unaffected by either factor, and no interaction was detected between turnout date and post grazing sward height. These findings suggest that maintaining a moderate post-grazing sward height of 5.5 cm provides more consistent benefits than tighter grazing to 3.5 cm, supporting grazing strategies that optimize sward height for improved efficiency and sustainability.

## 1. Introduction

Pasture-based beef production systems in temperate climates, such as in Ireland, are increasingly recognised for their capacity to incorporate high proportions of grazed and conserved herbage in animal diets, with increased herbage inclusion rates shown to enhance profitability [1]. Within such systems, grazed pasture is the most cost-effective feed resource [1,2]. Therefore, limiting dependence on high-cost feeds, such as concentrates, while optimising individual animal pasture live-weight gain and stocking rate, are essential for the economic sustainability of grass-based beef enterprises [3,4]. Beyond its effects on animal performance and grass utilisation, early turnout after winter housing has important economic implications for pasture-based ruminant systems. Earlier access to pasture reduces reliance on conserved forage in late winter and early spring, lowering winter silage requirements and associated feed costs. It also limits slurry accumulation during the housed period, reducing slurry storage demands and the labour, machinery, and contractor costs associated with slurry spreading. Collectively, these factors mean that even modest advances in turnout date can lead to meaningful reductions in variable costs at farm level, highlighting the value of management strategies that facilitate early turnout without compromising animal welfare or sward condition.

Reflecting the seasonality of pasture growth in temperate climates, most progeny are spring-born to match herd feed demands with pasture herbage supply [5]. In grass-based systems, calves from the dairy herd are typically spring born, reared indoors on milk replacer and limited concentrates, are typically weaned at around 10–12 weeks of age and then raised on graze pasture, with minimum concentrate supplementation [6]. At the end of the first grazing season, animals undergo an indoor ‘store’ period during the winter, where the diet primarily consists of grass silage with minimal supplementary concentrates. A second grazing season follows, which offers an opportunity to take advantage of compensatory growth [7] this is followed by a final indoor finishing period that incorporates grass silage and enhanced levels of concentrates, with slaughter typically occurring at 22–24 months of age [8]. The growing desire for ‘grass-fed’ beef has influenced beef systems to explore options to maximise weight gain at pasture and help to align production systems with consumer expectations of enhanced sustainability [9,10]. Reducing slaughter age has been identified as a means to reduce the carbon footprint in beef animals [3]. However, achieving a commercially acceptable carcass at a younger age can be challenging in dairy origin cattle, particularly those of Holstein Friesian breed due to their tendency for lower subcutaneous fat deposition compared to traditional beef breeds [11]. In rotational grazing systems, both pre- and post-grazing sward heights directly influence pasture yield and quality and to increase the proportion of grazed pasture in the diet an earlier turnout date to pasture is desirable. Taller pre-grazing swards offer more biomass but risk reducing herbage digestibility, while maintaining the optimal post-grazing sward height supports rapid regrowth and preserves forage nutritive value [12,13,14]. The refinement of grazing management strategies is therefore crucial to enhance pasture utilisation and animal performance within economically and environmentally sustainable production systems.

In contrast to the extensive guidelines developed for lactating dairy cows, optimal grazing strategies for dairy beef cattle remain less clearly defined [2,15]. Recommendations for dairy cows generally advise a post grazing sward height of 3.5–4 cm to optimise animal output per hectare, pasture production, and sward quality [5,16]. However, these targets can vary depending on the production system (e.g., spring calving vs. year-round calving), pasture type (e.g., perennial ryegrass vs. mixed swards), and prevailing climatic conditions. Although these ‘dairy-derived’ pasture management protocols are sometimes recommended for beef cattle [17], their direct application to dairy beef grazing systems is uncertain. Beef enterprises typically operate at much lower stocking rates than dairy farms (often <1.6 vs. >2.1 livestock units/ha), and concentrate supplementation during the grazing season, which is standard practice in dairy systems, is generally not considered an integral practice in beef production systems [1]. Adjustments to PGSH from approximately 4.0 to 6.0 cm have been shown to increase intake and growth in continental beef cattle, equivalent to approximately 30 kg liveweight gain over a 200-day grazing season [2,18,19].

To date, grazing management research focusing on varying turnout dates to pasture and PGSH has largely concentrated on traditional suckler systems, with emphasis on suckler cows [20], beef heifers [19], and continental beef breeds. However, limited attention has been given to dairy beef progeny, despite their increasing contribution to beef production systems [21,22,23]. While the advantages of early turnout to pasture for dairy cows are well established [24], there remains a scarcity of evidence concerning the potential medium to longer term benefits for dairy beef progeny.

Therefore, there is a clear need to determine grazing management guidelines specifically tailored to the biological and economic constraints of pasture-based dairy beef systems. In particular, evidence on how PGSH targets and turnout date affect animal performance and carcass traits of dairy and dairy × beef steers remains limited. Building on existing research, this study investigates whether grazing strategies, specifically variations in post-grazing sward height and turnout date, can be used to optimise performance of dairy and dairy × beef steers. Thus, the objectives of this study were to investigate the effects of spring turnout date and PGSH height on animal liveweight gain, dry matter intake, and carcass composition of dairy and dairy × beef steers reared under a grass-based Irish finishing system. It was hypothesised that earlier turnout to pasture and a higher post-grazing sward height would improve grazing-season performance through increased pasture intake, with potential carry-over effects on finishing performance and carcass traits.

## 2. Materials and Methods

### 2.1. Experimental Animals, Grazing Management, and Treatments

Three experiments were carried out at the Teagasc Grange Beef Research Centre over three production cycles using a total of 188 steers. Each treatment group was managed separately to maintain experimental integrity. Animal husbandry practices and methodological protocols were standardised across the studies to ensure consistency and comparability. The study included 60, 68, and 60 steers in Experiment 1, 2, and 3, respectively. In each production cycle, animals were managed in a calf-to-beef system from early life until slaughter at approximately 23–24 month of age. Calves were purchased at 3 weeks of age, reared indoors using established calf rearing practices until 10–12 weeks old, and then turned out to pasture, typically in May. The calves, grazed ahead (as Leaders) of older (Followers) animals in a Leader–Follower rotational grazing system. The Leader animals were housed for their first winter in November, accommodated in slatted floor sheds and offered grass silage ad libitum plus 1–2 kg concentrates/head/day. In March, these animals (now one year old) returned to pasture where they grazed their assigned pasture until the next calf crop were turned out in May, and from then onwards, the older animals graze as Followers with the younger calves grazing in the paddock immediately ahead of them.

The Followers were housed for finishing in mid- to late-October in their respective sub-groups (lying area = 2.58 m^2^/animal) and received an ad libitum grass silage plus concentrates (~5–6 kg fresh weight/head/day) diet until slaughter at ~23–24 months of age, producing a 280–300 kg carcass.

Experimental treatments were randomly assigned to dedicated paddocks, which were then rotationally grazed. Eight paddocks (~0.6 ha each) were allocated per treatment from turnout until mid-June, and were used sequentially within a rotational grazing system. These paddocks were not independent experimental units but were subdivisions of the grazing area used to manage pasture supply and maintain target post-grazing sward heights. Within each experiment, animals assigned to each treatment were managed as a single grazing group; however, individual animals within each treatment group were considered the experimental units for analysis.

Animal movement from one paddock to the next was dictated by the grass supply of the Followers. When the post-grazing sward height of the Followers reached the desired target (of either 3.5 or 5.5 cm), it triggered the movement of calves to their next paddock and for the Followers to move into the area vacated by the Leaders. Animals typically remained in a paddock for 2–4 days until the desired PGSH was obtained. The Leaders had an unrestricted pasture supply while grazing ahead of the Followers; therefore, the effect of the turnout date and post grazing sward height treatments was applied to the Following animals during their second grazing season. Animals received routine preventative treatments for internal and external parasites and were vaccinated against clostridial and respiratory diseases, based on best practice and veterinary advice. All calves were castrated by a veterinary surgeon during their first summer at pasture.

To produce sufficient winter feed for the animals, ~55% of the allocated farm area was harvested for first cut silage (in mid- to late-May), with an additional ~35% cut for a second time in early July. Following mowing for silage, the crop was wilted for up to 24 h and then picked up by a precision-chop harvester, and the crop thoroughly compacted in roofed, horizontal concrete silos, and sealed with black polythene sheeting weighted down with tyres. The mean values for dry matter, chemical composition, and in vitro digestibility of grass silage, grazed pasture, and supplementary concentrates offered during the indoor winter periods, across the three experiments, are summarised in Table 1. The animals grazed permanent (>20 year old) *Lolium perenne* dominant swards receiving ~120 kg chemical N/ha/annum and were stocked at ~2.7 livestock units (LU)/ha (220 kg organic N/ha). Swards cut for silage received 100 and 90 kg N/ha for first and second harvest, respectively, were typically 5–10 years old. Throughout the experimental period, all animals were supplied with unrestricted access to clean, fresh water.

Overall pasture supply on the grazed area was managed using the farm cover technique described by O’Donovan and Dillon [25], where sward heights were measured weekly on all paddocks available for grazing using a rising plate meter (Jenquip, Fielding, New Zealand) with 50 random readings taken per paddock and the resulting sward heights converted to dry matter yield/ha. Fifty random sward heights were recorded on each paddock before and after animal movements. This measurement technique was used to determine the pasture supply on each treatment and was used to estimate pre- and post-grazing herbage mass (kg DM/ha), as outlined by O’Riordan et al. [26]. In addition, pre- and post-grazing herbage yields were measured directly by cutting three representative strips (5.0 m long × 0.53 m wide) to a height of 3 cm using a rotary lawnmower (Honda HRX537VYEA, Honda Distributors, Dublin, Ireland). Collected herbage was weighed, dried overnight at 98 °C for dry matter determination. A further sample was dried at 48 °C for 48 h for laboratory analyses. Pre-grazing herbage, which exceeded ~3000 kg DM/ha, was removed from grazing and saved as round bale silage. The pasture disappearance method was employed to estimate daily grass dry matter intake (GDMI) of the experimental animals during their second grazing season [27]. GDMI was based on the dry matter yield difference between animals entering and leaving a paddock, accounting for group size, paddock area, and residency time.

All experimental procedures complied with the requirements of Experimental Licence B100/1099, in accordance with the Cruelty to Animals Act (1876) and the European Communities Regulations of 2002 and 2005.

### 2.2. Experiment 1—Animal Routine Management

Sixty male dairy × beef calves (Ho/Fr, Norwegian Red × HO/Fr and Jersey × Ho/Fr), with a mean (+/− standard deviation) birth date, age at purchase, and body weight of 29 March, 22 (s.d. 7.5) days and 52 (s.d. 5.8) kg, respectively, were sourced from commercial dairy farms. Calves were reared in a standard calf-rearing system at Teagasc Grange Research Centre, as summarised above before turnout to pasture in May. During their first grazing season, the calves grazed as Leaders in a rotational Leader–Follower system. After housing in November, the weanlings were individually penned and offered ad libitum grass silage supplemented daily with 1.5 kg/head/day coarse mixture until 20 January, after which the concentrates were withdrawn. Prior to the start of their second grazing season, animals were blocked by liveweight within breed and randomly assigned to one of four treatments in a 2 (turnout dates (TO): early, 13 March or late, 13 April) × 2 (post-grazing sward heights (PGSH): 3.5 or 5.5 cm) factorial arrangement of treatments. Each treatment group for each year was managed as a single grazing unit, and animals within each treatment group were considered the experimental units.

Animal stocking rate and grazing management were as described earlier. Liveweights were recorded at ~3-week intervals during the experimental period. During their second winter, steers were accommodated indoors on concrete slatted floor pens. Finishing management followed the common protocol (Section 2.1) and were offered an ad libitum grass silage supplemented with 5–6 kg concentrates/head/day diet.

### 2.3. Slaughter, Carcass Evaluation, and Tissue Collection

Animals were weighed prior to slaughter and transported ~130 km to a commercial abattoir for slaughter between 08:00 and 09:00 h. Post slaughter, the hide, feet, liver, and perirenal/retroperitoneal fat were weighed. Cold carcass weight was estimated at 98% of hot carcass weight, and kill-out proportion was calculated as cold carcass weight expressed as a proportion of pre-slaughter liveweight. Carcass conformation and fat score were recorded on a continuous 15-point scale [28]. The carcasses were split along the mid-line and chilled for 48 h. After 24 h in the chill, carcass length, chest depth, leg length, leg width, leg thickness, and round circumference of the right side of the carcass was measured using protocols described by McGee et al. [29].

After 48 h in the chill, the carcass right side was quartered between the 5th and 6th ribs into hind (pistola) and fore quarters. The cube roll was excised and dissected as per Dunne et al. [30], where ribs joint 6–10 were removed and weighed. The *longissimus* cross-section at the 10th rib was traced and the area measured using a digital planimeter (Placom KP-90N, Koizumi Sokki Co., Ltd., Tokyo, Japan). The rib section was dissected into subcutaneous fat, *M. longissimus*, other muscle, muscle trim, and bone (including *Ligamentum nuchae*), with each fraction weighed separately. A 7.5 × 7.5 cm section of subcutaneous fat was collected near the 10th rib. Fat colour (L*, a*, b*) was measured on the day of collection using a HunterLab UltraScan XE colourimeter (HunterLab, Reston, VA, USA).

Three 2.5 cm thick *longissimus thoracis et lumborum* (LT) steaks were taken, vacuum-packed, and transported to the Teagasc Food Research Centre, Ashtown and frozen. Upon later thawing, LT steaks were homogenised (Ultra-Turrax T25, IKA-Werke GmbH & Co. KG, Staufen, Germany), and intramuscular fat and moisture contents were assessed by microwave-assisted methylene chloride extraction [31]. Warner Bratzler shear force was measured following Wheeler et al. [32], using steaks thawed in a 10–15 °C circulating water bath and brought to room temperature before testing.

### 2.4. Experiment 2—Animal Routine Management

This study comprised of 34 Holstein–Friesian (HF) and 34 Aberdeen Angus × Holstein-Friesian (AA) crosses. Management of the calves during the first year was as described in Experiment 1. Prior to their second grazing season, the yearlings were blocked by weight from within breed and randomly assigned to one of four treatments, in a 2 (early turnout: ET, 8 March or late turnout: LT, 31 March) × 2 (post-grazing sward height (PGSH: 3.5 or 5.5 cm) factorial arrangement of treatments. Each treatment comprised 17 animals managed as a single experimental unit. Treatments were applied at the grazing group level. Individual animals within each treatment group were considered the experimental units for liveweight, growth, and carcass measurements, while intake was analysed at the group level.

Grazing and animal management was as described earlier. Animals were weighed approximately every three weeks and housed on 15 October. Similar to Experiment 1, at the end of the grazing season, the steers were accommodated indoors in concrete slatted-floor pens and fed as described in Section 2.1.

Throughout the indoor finishing phase, steers had unrestricted access to grass silage and received a daily allocation of 6 kg concentrates (fresh weight)/head until slaughter in mid-March. Animals were weighed before slaughter and subsequently transported approximately 130 km to a commercial abattoir early morning, where slaughter commenced shortly after arrival. Cold carcass weight determination was as described previously. Carcass conformation and fat scores were assessed using a continuous 15-point scale, according to the EU beef carcass classification system as described by Conroy et al. [28].

### 2.5. Experiment 3—Animal Routine Management

In a third study, sixty yearling steers (similar calf rearing protocols and pasture management as per Experiments 1 and 2) comprising 30 Belgian Blue × Holstein–Friesian (BB) and 30 Aberdeen Angus × Holstein-Friesian (AA) were used. Prior to their second grazing season, the animals were blocked by liveweight from within breed and randomly assigned to one of four treatments in a 2 (early turnout, ET, 23 March or late turnout, LT, 12 April) × 2 (post-grazing sward height (PGSH: 3.5 or 5.5 cm) factorial arrangement of treatments. Each treatment comprising 15 animals per treatment was managed as a single grazing group. Grazing protocols and animal management was as described earlier. Similar to Experiments 1 and 2, at the end of the grazing season, steers were accommodated indoors in concrete slatted floor pens. Throughout the finishing phase, steers had unrestricted access to grass silage and received a daily allocation of 6 kg/head (fresh weight) of concentrates. Finishing management, slaughter procedures, and carcass evaluation for Experiment 3 were conducted in accordance with the established protocols used in Experiment 1 and 2 to maintain consistency across all treatment groups.

### 2.6. Statistical Analysis

The animal experimental data were analysed using SPSS (Statistical Package for the Social Sciences, version 27), with each experiment analysed separately. Treatments (turnout date and post-grazing sward height) were applied at the level of the grazing group. Individual animals within each treatment group were considered the experimental units. Animal-level data (e.g., liveweight, growth, carcass traits) were analysed using mixed models to account for within-group variation; however, inference on treatment effects was based on consistency across independent production cycles. Prior to analysis, normality of residuals was assessed using graphical methods, including box plots and residuals versus fitted value plots, and homogeneity of variances was evaluated using Levene’s test. For animal-level traits, data were analysed using the MIXED procedure, with turnout date (early vs. late), post-grazing sward height (PGSH), and their interaction included as fixed effects, and animal ID specified as a random effect. Repeated liveweight measurements included time as a repeated factor with animal ID as the subject. Average daily gain (ADG) was calculated as the slope of the linear regression of liveweight (kg) on time (days) and used as the response variable in subsequent analyses. Group-level traits (e.g., dry matter intake) were analysed using the General Linear Model (GLM) procedure, with treatment as a fixed effect. Post hoc comparisons were performed using Tukey’s HSD test. Results are presented as least-squares means (LSMean) ± standard error of the mean (SEM). To account for the grouped structure of the data, a year-means ANOVA was also conducted, where each Year × Turnout × PGSH combination was treated as a single observational unit, with year included as a blocking factor. This approach provided independent replication of treatment effects across the three experiments. Breed was included as a fixed effect, and breed × treatment interactions were tested but were not retained in the final model due to lack of significance. Differences were considered significant at *p* < 0.05 and trends at 0.05 < *p* < 0.10.

## 3. Results

Rainfall for the grazing season (March–November) was 46% and 4% greater than the long-term average in Experiment 1 and 2, respectively, while Experiment 3 received 8% less rainfall. Mean 5 cm depth soil temperatures for the same period were 12.6, 10.7, and 11.5 °C in Experiments 1, 2, and 3, respectively, compared with a 50-year average of 11.0 °C.

Mean pre-grazing herbage masses for 3.5 cm and 5.5 cm swards were 1250 and 1283 kg DM/ha, 1421 and 1520 kg DM/ha, and 1355 and 1475 kg DM/ha in Experiment 1, 2, and 3, respectively. Corresponding mean post-grazing residuals averaged 113 and 156 kg DM/ha in Experiment 1, 142 and 212 kg DM/ha in Experiment 2, and 131 and 159 kg DM/ha in Experiment 3.

### 3.1. Experiment 1: Animal Growth and Intake

While the early turnout was associated with improved liveweights early in the grazing season, the effect was not significant at housing nor at slaughter (Table 2). Neither average daily gain at pasture or during the winter, nor carcass weight, conformation, and fat scores or KO% were affected by turnout date. However, carcass weight per day of age tended (*p* < 0.090) to be higher for the early turnout animals.

Grazing swards to a stubble height of 5.5 cm was associated with higher animal gains at pasture (*p* = 0.001) and a 34 kg heavier housing weight (*p* = 0.005); carcass weight and kill-out percentage were numerically higher at 5.5 cm but were not statistically different (carcass weight *p* = 0.498; kill-out *p* = 0.199).

However, animal grazing swards to 3.5 cm had higher indoor finishing gains (*p* < 0.006). Carcass conformation or fat scores were unaffected by either turnout date or post-grazing sward height.

Animal intake during finishing was unaffected by either turnout date or post-grazing sward height. There was no interaction between animal breed and turnout date nor between animal breed and post-grazing sward heights. Performance of the Leader calves was unaffected by the post-grazing swards treatments applied to the Followers.

#### 3.1.1. Body Measurements, Ultrasonic Measurements and Non-Carcass Components

Carcass physical measurements and ultrasonic muscle and fat depth were unaffected by turnout date (Table 3). Of the offal components measured, only liver and perirenal and retroperitoneal fat were affected, being higher (*p* < 0.05) for the early turnout animals.

Back length was higher (*p* < 0.01) and pelvic width lower (*p* < 0.05) for animals grazed to 5.5 cm. Other carcass measurements were unaffected by post-grazing sward height. There was an interaction between turnout date and post-grazing height for back length, and it occurred where steers grazed to 5.5 cm had longer backs, compared with those at 3.5 cm, and this effect was more pronounced in late turnout animals (TO × PGSH, *p* = 0.007).

#### 3.1.2. *M. longissimus* Area, Rib Joint Composition, and Adipose Tissue Colour

With the exception of fat weight in the cube roll, which was higher (*p* < 0.05) for the early turnout animals, neither turnout date nor post-grazing sward height had an effect on *M. longissimus* area, nor rib composition (Table 4). Carcass adipose lightness, redness, or yellowness (L*, a*, and b* values, respectively), were not affected by treatment.

Post-grazing sward height had no effect on muscle lipid, moisture, protein content, shear force, or pH values (*p* > 0.05) (Table 5). Muscle lipid content was higher (*p* < 0.05) and moisture content lower (*p* < 0.05) in early turnout steers, while protein, shear force, and muscle pH were unaffected (*p* > 0.05). A significant interaction between turnout date and post-grazing sward height was observed for muscle lipid, with early turnout steers grazed to 5.5 cm having the highest lipid content and late turnout steers grazed to 3.5 cm the lowest.

There was no interaction between animal breed and turnout date nor between breed and post-grazing sward heights. Calf performance, grazing as Leaders, was unaffected by the post-grazing swards treatments applied to the Followers.

### 3.2. Experiment 2

#### 3.2.1. Animal Growth and Pasture Intake

Apart from minor liveweight differences up to early May, when early turned out animals tended to be heavier, turnout date did not result in significantly different liveweights at housing (*p* > 0.05) (Table 6). Liveweight gain at pasture was likewise unaffected by turnout date (*p* > 0.05). Estimated pasture intake was similar for both turnout dates (*p* > 0.05).

Post-grazing sward height of 5.5 cm was associated with higher (~25 kg) housing weight (*p* < 0.05), and animals managed at the higher sward height achieved a greater average daily gain during the grazing period (*p* < 0.001) and was accompanied by a higher pasture DM intake (*p* < 0.001). However, finishing-period gains did not differ between sward-height treatments (*p* > 0.05).

#### 3.2.2. Slaughter and Carcass Characteristics

Slaughter data and carcass weights, carcass conformation and fatness and kill-out% are summarised in Table 7, and these variables were not affected by turnout date nor post-grazing sward height.

### 3.3. Experiment 3

#### 3.3.1. Animal Growth and Pasture Intake

Earlier turnout initially resulted in lower liveweights (*p* < 0.05), but by early June, weight differences were not significantly different (*p* > 0.05) (not shown). Average daily gains from mid-April to early September were higher for the early turnout animals (*p* < 0.05); however, housing liveweight and subsequent winter weights were unaffected by turnout date (*p* > 0.05) (Table 8). The effects of post-grazing sward height on early grazing season live weights were small, but overall average daily gains from mid-April to late June and from mid-April to early September were higher on the 5.5 cm post-grazing sward height treatment (*p* < 0.05). At housing, there was a 28 kg liveweight difference in favour of the more laxly grazed animals (*p* < 0.05). Post housing, animals previously grazed to the lower sward height had higher (*p* < 0.05) daily gains. Pasture intake was similar for both turnout dates but was higher (*p* < 0.05) for the animals having the higher post-grazing sward height. Indoor silage intakes during the finishing period in Experiment 3 were not affected by either turnout date or PGSH.

#### 3.3.2. Slaughter and Carcass Characteristics

Slaughter and carcass weights were not significantly affected by either turnout date or post-grazing sward height (*p* > 0.05, Table 9). Likewise, neither carcass conformation and fat score or kill-out% or carcass weight per day of age were affected by either turnout date or post-grazing sward height treatments.

As observed in Experiment 1 and 2 there was no interaction between breed and turnout date or between breed and post-grazing sward heights. Performance of the Leader calves was unaffected by treatment. In addition, the year-means ANOVA conducted to guard against pseudo-replication supported the main treatment effects observed in the animal-level analyses, with no changes in the overall interpretation of the results.

## 4. Discussion

This study, involving 188 steers of dairy and dairy × beef origin, evaluated the effects of spring turnout date and post-grazing sward height on animal performance in a pasture-based system. Through the assessment of liveweight gain, pasture intake, carcass traits, and meat quality, the study provides insight into how defined adjustments to grazing management can influence growth performance and carcass outcomes in temperate grassland systems. Maximising the contribution of grazed pasture remains central to the competitiveness of beef production in temperate climates and continues to represent the most economical feed resource available [33]. This work contributes to understanding grazing management in dairy-beef systems by quantifying the effects of turnout date and post-grazing sward height on animal performance and carcass characteristics under pasture-based conditions. It is important to note that, as treatments were applied at the grazing group level, the study does not include within-year treatment replication, and findings should therefore be interpreted as descriptive patterns across years rather than definitive treatment effects.

### 4.1. Turnout Date

Compared with post-grazing sward height, turnout date exerted limited and short-lived effects on steer performance. Early turnout steers achieved approximately 90–110 g/day higher ADG than late turnout groups during the early grazing season; however, these differences were not maintained beyond mid-season and did not result in differences at housing or slaughter. Across all experiments, turnout date had no effect on liveweight at housing, finishing-period gain, slaughter weight, or carcass traits, including conformation, fat score, kill-out percentage, and carcass weight per day of age. Similar short-term improvements following early turnout have been reported by Walsh et al. [24], where increased performance was attributed to the higher digestibility and energy content of early-season pasture. In contrast, Kennedy et al. [34] reported more sustained responses in dairy systems, likely reflecting the higher nutrient demand associated with lactation. The present findings, therefore, indicate that, in dairy–beef systems, early performance advantages are transient and do not persist to final production outcomes. The lack of sustained turnout effects is also consistent with compensatory growth responses, whereby animals recover from early differences during the finishing period. Similar recovery patterns have been reported by Keady et al. [7], supporting the convergence of liveweight and carcass characteristics observed in the present study. Furthermore, broader grazing-systems research shows that stocking rate and pasture utilisation efficiency exert stronger influences on animal performance than turnout date alone, supporting the limited effects observed here. [35]. In addition, the turnout interval differed across experiments (31, 23, and 20 days), and the narrower interval in Experiments 2 and 3 may have reduced treatment contrast and contributed to the weaker turnout effects observed. From a biological perspective, the initial increase in ADG following early turnout is likely driven by improved pasture quality and increased intake as animals transition from winter diets to high-digestibility spring herbage. However, as grazing conditions become more uniform later in the season, these advantages diminish. Short-term changes around turnout are also consistent with gut-fill adjustment when cattle transition from silage to pasture [36]. Any associated liveweight dips were temporary, typically resolving within 14–21 days, and did not influence subsequent performance. Because such responses can vary with weather, pasture supply, and measurement timing, grazing outcomes are best interpreted at a seasonal rather than weekly scale [24,37].

Overall, turnout date exerted a small and transient influence on performance compared with the stronger effects of post-grazing sward height.

### 4.2. Post-Grazing Sward Height

Across the three studies, a consistent pattern emerged in which steers grazed at the more lax post-grazing sward height (PGSH) of 5.5 cm achieved higher growth rates throughout the grazing season. Liveweight advantages became evident early in spring and widened steadily until housing in mid-October, amounting to differences of 34, 25, and 28 kg in Experiments 1, 2, and 3, respectively. These results reinforce the principle that maintaining sufficient pasture height improves dry-matter intake, bite mass, and grazing efficiency, thereby supporting superior liveweight gain during the grazing season [2,19,21], and are consistent with broader evidence that higher residuals (lower grazing intensity) enhance individual animal performance [38]. Similar reductions in per-animal performance under more severe grazing residuals have been documented in controlled rotational grazing studies [39]. In contrast, steers grazed to 3.5 cm consistently exhibited higher indoor ADG during the finishing period. By slaughter, liveweight differences between PGSH treatments were reduced to 12, 15 and 12 kg for Experiments 1, 2, and 3, respectively. Compensatory growth was evident during finishing in steers previously grazed to 3.5 cm, where 65, 40, and 57% of the liveweight differences at housing were recovered by slaughter. This response is consistent with compensatory growth patterns reported in beef cattle [7], where increased growth rates during re-alimentation are driven by improved feed efficiency and nutrient utilisation following earlier restriction. The variation in recovery across experiments may reflect differences in the magnitude of initial liveweight differences at housing, as well as variation in grazing conditions and finishing duration. Previous research has shown that the extent of compensatory growth is influenced by the severity and duration of nutritional restriction, with moderate differences in liveweight often resulting in partial rather than complete recovery [7]. In the present study, the relatively moderate liveweight differences at housing (25–34 kg) likely contributed to the incomplete compensation observed. Despite improved finishing gains in steers grazed to 3.5 cm, carcass weight and composition were not significantly affected by PGSH. Carcass traits including conformation, fat class, kill-out percentage, and carcass weight per day of age remained consistent across treatments when slaughtered at approximately 600–640 kg liveweight, producing carcass weights of 290–312 kg [18,21]. This indicates that differences in grazing-season performance largely influenced the distribution of growth rather than final carcass outcomes, a pattern also reported in previous grazing studies [2,19]. In effect, higher gains achieved at pasture under greater PGSH were largely offset by increased finishing performance in the lower-PGSH group, limiting any divergence at slaughter [21,40]. Across all three grazing years, pre-grazing herbage mass remained within the normal range expected for Irish rotational grazing systems [25,26], indicating that differences in performance were driven by grazing management rather than by variation in pasture availability. These patterns are consistent with pasture-growth principles demonstrating that maintaining adequate post-grazing leaf area supports both intake and regrowth, sustaining sward productivity and animal performance across rotations [41].

### 4.3. Meat Quality and Organ Weights

Meat quality traits measured in Experiment 1 including ultimate pH, colour (L*, a*, b*) and shear force were not affected by turnout date or post-grazing sward height, indicating that moderate adjustments to grazing management did not compromise the eating quality of beef produced. This aligns with previous work showing that forage-based systems can deliver consistent meat quality when appropriate sire lines and finishing strategies are used [22,42,43,44]. Small compositional differences were detected: early turnout was associated with slightly higher intramuscular lipid and lower moisture, and some internal organ weights (e.g., liver, perirenal/retroperitoneal fat) also differed between treatments. However, these differences should be interpreted cautiously because turnout dates differed by only ~3 weeks, sample sizes for organ measurements were limited, and neither PGSH nor carcass traits showed corresponding effects. Taken together, these patterns are more consistent with normal biological variation than with strong treatment-driven responses [45,46]. In line with established carcass composition principles, structural tissues such as bone, hide, and feet remained stable across treatments and are typically influenced by breed and age rather than short-term grazing management [23,47,48].

Overall, the data indicate that meat quality was robust to the moderate grazing differences imposed in this study, and that observed compositional variation was minor and unlikely to be of practical significance.

### 4.4. Overview

One of the key observations from this study was the absence of a turnout × PGSH interaction for animal performance, carcass traits, and meat quality. This indicates that, under the conditions of the present study, the effects of turnout date and post-grazing sward height were largely additive. However, this finding should be interpreted with caution, as the absence of a statistically significant interaction does not necessarily confirm independence between factors. These results nonetheless suggest that both turnout date and post-grazing sward height can be considered as separate management factors within the range of conditions evaluated, each contributing differently to animal performance across the production cycle [49,50]. In particular, post-grazing sward height had a more consistent influence on grazing-season performance, while turnout date primarily affected early-season growth. From a systems perspective, this indicates some level of flexibility in grazing management, although outcomes will depend on environmental conditions, pasture availability, and overall system design [9,49,50]. Consequently, the relative importance of turnout date and post-grazing sward height should be considered within the broader seasonal and management context rather than as fully independent drivers of performance and aligning grazing to periods of strong pasture growth further optimises pasture utilisation and supports more consistent animal performance, as shown in pasture-focused studies [16,51]. Decision-support tools such as PastureBase Ireland [35] can help apply these findings in real time by tracking pasture availability, guiding turnout timing, and monitoring growth across seasons.

## 5. Conclusions

This study demonstrates that moderate adjustments in grazing management can influence the distribution of growth across the production cycle without altering final carcass outcomes. Across all experiments, earlier turnout produced small, non-significant and short-lived improvements in early-season liveweight, but these differences did not persist to housing or slaughter. In contrast, maintaining a higher post-grazing sward height (5.5 cm) consistently supported greater pasture intake and higher grazing-season gains, although compensatory growth in the 3.5 cm group during finishing reduced but did not remove these differences. Carcass weight, conformation, fat class, and kill-out percentage were similar across all treatments, indicating that PGSH affected when growth occurred rather than how much was ultimately produced.

No interaction between turnout date and PGSH was detected, suggesting that these factors can be adjusted independently under comparable conditions. From a practical perspective, maintaining adequate residual sward height is a more reliable strategy for improving grazing-season performance than altering turnout date alone. Earlier turnout may still offer management value where weather permits substitution of indoor feed with pasture, even if biological responses are modest. Future research should explore genotype-specific responses, alternative sward types and the role of precision grazing technologies to refine grazing management in pasture-based beef systems.

## Figures and Tables

**Table 1:** Mean feed analysis across grazing and finishing periods for 3 experiments.

Period/Feed	Component	Value	SD	Units
First winter silage	Dry matter (DM)	198	±12	g/kg
	Digestible dry matter (DMD)	692	±18	g/kg
	Crude protein (CP)	133	±9	g/kg
	Ash	99	±6	g/kg
	pH	3.9	±0.10	—
	UFV	0.77	±0.04	unit/kg DM
Grazing herbage	Dry matter (DM)	188	±14	g/kg
	Crude protein (CP)	177	±12	g/kg
	Digestible dry matter (DMD)	717	±20	g/kg
Second winter silage	Dry matter (DM)	189	±10	g/kg DM
	Crude protein (CP)	148	±8	g/kg DM
	Ash	89	±5	g/kg DM
	Neutral detergent fibre (NDF)	580	±25	g/kg DM
	Acid detergent fibre (ADF)	374	±18	g/kg DM
	In vitro DMD	745	±22	g/kg DM
	pH	4.1	±0.08	—
Finishing concentrate	Dry matter (DM)	770	±18	g/kg
	Neutral detergent fibre (NDF)	146	±10	g/kg DM
	Crude protein (CP)	144	±6	g/kg DM
	UFV	1.22	±0.05	

**Table 2:** Impact of spring turnout date and post-grazing sward height on performance and carcass characteristics of steers (Experiment 1).

	TURNOUT				PGSH			
*Liveweight* (kg)	Early	Late	SEM	*p*-Value	3.5	5.5	SEM	*p*-Value
Start of trial	275.0	276.0	7.60	0.369	275.6	275.3	7.60	0.665
Housing weight	405.2	398.9	8.20	0.178	383.0	417.0	8.20	0.005
Final liveweight at slaughter	601.03	592.0	10.40	0.318	593.7	594.2	10.40	0.913
*ADG* kg/day								
Turnout to housing	0.73	0.71	0.025	0.486	0.656	0.787	0.025	0.001
Housing to slaughter	1.02	1.07	0.07	0.576	1.15	0.94	0.074	0.006
Carcass weight per day of age (g)	0.409	0.388	0.006	0.090	0.439	0.394	0.006	0.477
Carcass weight (kg)	291.7	286.7	6.21	0.750	287.1	290.5	6.21	0.498
Kill-out (g/kg)	48.55	48.93	0.34	0.613	48.36	48.90	0.30	0.199
Carcass conformation class	4.03	4.00	0.25	0.612	4.10	3.93	0.25	0.639
Carcass fat class	9.13	8.66	0.21	0.876	8.93	8.86	0.21	0.693
Indoor feed intakes (kg DM/head/day)	10.65	10.88	0.25	0.572	10.94	10.61	0.254	0.406

*p*-values for Final LW, finishing ADG, and carcass weight were derived from models including slaughter date as a fixed effect. SEM = standard error of the mean. PGSH = post-grazing sward height. No significant TO × PGSH interaction were observed.

**Table 3:** Effects of turnout date and post-grazing sward height on body dimensions, tissue composition, and organ weights of steers (Experiment 1).

	TURNOUT				PGSH				
	Early	Late	SEM	*p*-Value	3.5	5.5	SEM	*p*-Value	TO × PGSH
*Body measurements* (cm)									
Withers height	129.8	130.0	0.85	0.87	129.6	130.2	0.85	0.62	0.91
Chest girth	172.4	172.1	6.10	0.98	171.7	172.8	6.10	0.89	0.90
Chest depth	69.7	69.5	0.55	0.80	69.4	69.8	0.55	0.55	0.390
Back length	126.2	124.5	0.95	0.22	123.6	127.0	0.95	0.01	0.007
Pelvic width	46.4	46.6	0.61	0.82	47.4	45.6	0.61	0.04	0.940
*Ultrasonic measurements* (mm)									
Fat depth	3.1	3.5	0.32	0.46	3.6	3.0	0.32	0.33	0.80
Muscle depth	54.8	55.0	0.96	0.90	54.9	55.0	0.96	0.94	0.701
*Weight* (kg)									
Hide	38.7	38.4	1.0	0.72	39.3	37.8	1.0	0.30	0.36
Fore feet	5.4	5.4	0.10	0.71	5.4	5.4	0.10	0.79	0.855
Hind feet	5.4	5.7	0.14	0.83	5.34	5.4	0.14	0.91	0.847
Liver	7.6	7.1	0.151	0.02	7.3	7.8	0.151	0.75	0.844
Perirenal and retroperitoneal fat	11.8	9.7	0.622	0.019	11.5	10.1	0.623	0.12	0.392

SEM = standard error of the mean. PGSH = post-grazing sward height.

**Table 4:** Effects of turnout date and post-grazing sward height on muscle area, rib joint composition, and adipose tissue colour for dairy beef steers (Experiment 1).

	TURNOUT				PGSH			
	Early	Late	SEM	*p*-Value	3.5	5.5	SEM	*p*-Value
*M. longissimus* (cm^2^)	59.35	56.88	1.6	0.278	57.83	58.41	1.65	0.797
*M. longissimus* (cm^2^/kg carcass)	0.202	0.204	0.005	0.907	0.203	0.201	0.0054	0.761
*Rib joint composition* (kg)								
Rib total	6.71	6.47	0.11	0.126	6.65	6.52	0.11	0.404
Muscle	4.25	4.12	0.082	0.149	4.21	4.12	0.082	0.417
*Fat*	1.50	1.42	0.031	0.047	1.48	1.43	0.031	0.169
Bone	0.95	0.93	0.041	0.214	0.947	0.940	0.0415	0.655
*Adipose colour*								
*Lightness*	61.94	61.74	0.154	0.660	62.039	61.74	0.154	0.177
Redness	10.55	10.72	0.081	0.224	10.51	10.69	0.081	0.471
Yellowness	17.10	17.30	0.105	0.456	17.17	17.21	0.105	0.772

SEM = standard error of the mean. PGSH = post-grazing sward height. No significant TO × PGSH interaction were observed.

**Table 5:** Effects of turnout date and post-grazing sward height on meat composition and quality attributes for steers (Experiment 1).

	TURNOUT				PGSH				
Composition (g/kg)	Early	Late	SEM	*p*-Value	3.5	5.5	SEM	*p*-Value	TO × PGSH
Lipid	6.87	5.24	0.534	0.032	5.95	6.12	0.534	0.833	0.020
Moisture	70.40	71.86	0.414	0.019	71.38	70.91	0.414	0.482	0.475
Protein	21.78	21.75	0.412	0.983	21.78	21.77	0.412	0.967	0.306
Shear Force	29.11	29.70	0.311	0.184	29.43	29.38	0.311	0.905	0.389
pH	5.58	5.61	0.031	0.851	5.59	5.61	0.031	0.851	0.546

SEM = standard error of the mean. PGSH = post-grazing sward height.

**Table 6:** Effects of turnout date and post-grazing sward height on animal performance and pasture intake (Experiment 2).

	TURNOUT				PGSH			
*Liveweight* (kg)	Early	Late	SEM	*p*-Value	3.5	5.5	SEM	*p*-Value
Start of trial	256.7	256.6	3.21	0.994	256.7	256.6	8.21	0.994
Housing weight	477.6	476.1	5.7	0.899	462.0	487.2	5.70	0.012
Final liveweight at slaughter (kg)	608.6	614.2	7.3	0.703	603.5	619.3	7.3	0.281
*ADG* kg/day								
Turn out to housing	0.71	0.70	0.020	0.712	0.64	0.77	0.020	0.001
Housing to slaughter	1.07	1.13	0.040	0.455	1.14	1.06	0.040	0.279
Pasture Intakes (kg DM/head/day)	6.73	6.72	0.070	0.964	6.51	7.24	0.070	0.001

SEM = standard error of the mean. PGSH = post-grazing sward height. No significant TO × PGSH interaction were observed.

**Table 7:** Effects of turnout date and post-grazing sward height on carcass characteristics of beef steers. Experiment 2.

	TURNOUT				PGSH			
	Early	Late	SEM	*p*-Value	3.5	5.5	SEM	*p*-Value
Carcass weight	303.3	312.4	4.66	0.336	308.9	311.8	4.66	0.398
Conformation	4.80	5.04	0.273	0.561	4.71	5.14	0.273	0.260
Fat class	8.19	8.22	0.29	0.955	8.01	8.40	0.29	0.344
Kill-out %	50.03	49.55	0.44	0.441	49.6	49.98	0.44	0.557
Carcass weight per day (g)	0.427	0.423	0.010	0.360	0.428	0.422	0.010	0.325

SEM = standard error of the mean. PGSH = post-grazing sward height. No significant TO × PGSH interactions were observed.

**Table 8:** Effects of turnout date and post-grazing sward height on animal performance and pasture intake in steers (Experiment 3).

	TURNOUT				PGSH			
*Liveweight* (kg)	Early	Late	SEM	*p*-Value	3.5	5.5	SEM	*p*-Value
Start of trial	285.3	285.5	4.97	0.984	285.4	285.3	4.97	0.981
Housing weight	432.7	433.5	7.20	0.937	418.9	447.3	7.20	0.007
Final liveweight	620.0	636.7	14.75	0.330	623.5	633.3	14.75	0.642
*ADG* kg/day								
Turnout to housing	0.71	0.70	0.026	0.244	0.64	0.78	0.026	0.001
Housing to slaughter	1.03	1.08	0.025	0.442	1.13	0.99	0.028	0.025
Pasture intakes (kg DM/head/day)	6.77	6.71	0.30	0.172	6.45	7.27	0.30	0.001

SEM = standard error of the mean, PGSH = post-grazing sward height. Note. No significant TO × PGSH interaction was observed.

**Table 9:** Effects of turnout date and post-grazing sward height on slaughter metrics and carcass characteristics for dairy cross beef steers (Experiment 3).

	TURNOUT				PGSH			
	Early	Late	SEM	*p*-Value	3.5	5.5	SEM	*p*-Value
Slaughter weight	620.0	636.7	14.75	0.432	623.5	633.3	14.75	0.642
Carcass weight	312.5	307.5	6.65	0.330	315.2	306.5	6.65	0.390
Conformation	6.13	6.09	0.221	0.596	6.20	6.03	0.221	0.911
Fat class	8.70	9.14	0.27	0.215	8.76	9.07	0.27	0.386
Kill-out %	51.85	49.12	2.55	0.421	51.7	49.03	2.55	0.459
Carcass weight per day (g)	0.426	0.414	0.016	0.638	0.429	0.411	0.016	0.485

SEM = standard error of the mean. PGSH = post-grazing sward height. No significant TO × PGSH interaction were observed.

## Data Availability

The datasets used and analysed during the current study are available from the corresponding author upon reasonable request.
